# Intra-articular injection of kartogenin promotes fibrocartilage stem cell chondrogenesis and attenuates temporomandibular joint osteoarthritis progression

**DOI:** 10.3389/fphar.2023.1159139

**Published:** 2023-06-09

**Authors:** Jin Qiu, Bingqiang Hua, Xiaoping Ye, Xianwen Liu

**Affiliations:** Department of Oral and Maxillofacial Surgery, Stomatological Hospital, Southern Medical University, Guangzhou, China

**Keywords:** temporomandibular joint osteoarthritis, kartogenin, cartilage, fibrocartilage stem cells, chondrogenic differentiation

## Abstract

**Introduction:** Kartogenin (KGN) is a small-molecule compound that has been reported to improve the chondrogenic differentiation of mesenchymal stem cells *in vitro* and to alleviate knee joint osteoarthritis in animal models. However, whether KGN has any effect on temporomandibular joint osteoarthritis (TMJOA) remains unclear.

**Methods:** We first performed partial temporomandibular joint (TMJ) discectomy to induce TMJOA in rats. Histological analysis, tartrate-resistant acid phosphatase staining, and immunohistochemistry were used to assess the therapeutic effect of KGN on TMJOA *in vivo*. CCK8 and pellet cultures were used to determine whether KGN treatment could promote the proliferation and differentiation of FCSCs in vitro. Quantitative real-time polymerase chain reaction (qRT-PCR) was conducted to determine the expression of aggrecan, Col2a1, and Sox9 in FCSCs. Furthermore, we performed western blot to analysis the effect of KGN treatment on the expression of Sox9 and Runx2 in FCSCs.

**Results and discussion:** Histological analysis, tartrate-resistant acid phosphatase staining, and immunohistochemistry showed that intra-articular injection of KGN attenuated cartilage degeneration and subchondral bone resorption in vivo. Further analyses of the underlying mechanisms revealed that KGN enhanced chondrocyte proliferation, increased the number of cells in both superficial and proliferative zones of TMJ condylar cartilage *in vivo*, enhanced the proliferation and chondrogenic differentiation of fibrocartilage stem cells (FCSCs), and upregulated the expression of chondrogenesis-related factors *in vitro*. Collectively, in our study, KGN was shown to promote FCSC chondrogenesis and restore TMJ cartilage, suggesting that KGN injections might be a potential treatment for TMJOA.

## 1 Introduction

Temporomandibular joint osteoarthritis (TMJOA) is a chronic disease characterized by progressive cartilage degeneration, subchondral bone remodeling, synovitis, and pain (Zarb & Carlsson, 1999; Machon et al, 2017). Currently, TMJOA management is largely based on disease severity. For early- or mid-stage patients, conservative treatments, such as medication, splints, intra-articular injections, joint punctures, or arthroscopic surgery, are preferred. Patients with severe disease are usually treated via more radical treatment methods, such as open joint surgery, yet these often fail or cause further damage (Ouanounou et al, 2017; Haigler et al, 2018; Zhang et al, 2019; Zhao & Xie, 2021). Therefore, great efforts have been made to develop advanced treatment strategies for TMJOA. In recent years, stem cell-based therapy has attracted wide attention and is considered a promising therapeutic strategy for TMJOA. Currently, exogenous stem cell implantation and endogenous stem cell recruitment are the two main stem cell-based therapies being explored (Miller & Kaplan, 2012). However, the feasibility of exogenous stem cell transplantation depends on overcoming a number of obstacles, such as immune rejection, pathogen transmission, and potential tumorigenesis (Pelttari et al, 2014; Shen et al, 2014; Zhang et al, 2015). It is now recognized that tissue-resident stem cells maintain homeostasis and, in some instances, facilitate repair, with their depletion or dysfunction potentially accelerating tissue aging due to abnormal repair (Su et al, 2009; Wagers, 2012; Chandel et al, 2016). These findings have given rise to the idea that the use of endogenous stem cells rather than exogenous stem cell transplantation may be more advantageous for the repair and maintenance of damaged condylar cartilage homeostasis (Miller & Kaplan, 2012). In some cases, therapeutic strategies that recruit endogenous stem cells might eliminate the need for stem cell-based transplantation and all of the complications that accompany it.

Fibrocartilage stem cells (FCSCs) localized within the superficial zone niche in the temporomandibular joint (TMJ) condyle harbor considerable chondrogenic as well as osteogenic potential and can be used for the regeneration of cartilage and joint injury resolution (Embree et al, 2016). When FCSCs are transplanted into bone defects, intramembranous bone can be engrafted and regenerated (Nathan et al, 2018). FCSCs have also been found in human TMJ cartilage, having greater chondrogenic capacity than human orofacial mesenchymal stem cells (Bi et al, 2020). Furthermore, current studies have demonstrated that FCSCs can, through a variety of mechanisms, support the maintenance of TMJ homeostasis and the recovery of articular cartilage and subchondral bone in inflammatory conditions (Ruscitto et al, 2020; Bi et al, 2022). The identification of FCSCs would enable the harnessing for cartilage repair during TMJ-cartilage degeneration.

Previous reports revealed that the small molecule kartogenin (KGN) can stimulate the proliferation and chondrogenic differentiation of mesenchymal stem cells (MSCs) by regulating the CBF-RUNX1 transcriptional program (Johnson et al, 2012). When KGN was injected into an intact rat patellar tendon *in vivo*, cartilage-like tissue formation was induced in the injection area (Zhang & Wang, 2014). During limb development in mice, KGN also stimulated cartilage nodule formation by upregulating genes encoding hedgehog and TGF-β1 superfamily members, particularly TFG-β1 (Decker et al, 2014). In recent years, many studies have demonstrated that KGN promotes cartilage regeneration by inducing the differentiation of endogenous stem cells (Decker et al, 2014; Zhang & Wang, 2014; Im, 2018; Liu et al, 2020; Zeng et al, 2021). However, whether KGN could promote the chondrogenic differentiation of FCSCs and thus repair degenerated TMJ cartilage remains unknow.

Therefore, the purpose of this study was to investigate the therapeutic effect of KGN on TMJOA and the role of KGN in promoting the chondrogenic differentiation of FCSCs. In the present study, we first established a rat TMJOA model *in vivo* via partial discectomy and then extracted FCSCs from the surface of the rat TMJ condyle for *in vitro* experiments to study the therapeutic effect of KGN on TMJOA and its underlying mechanism. This study extends our understanding of the effect of KGN on FCSCs and may provide new avenues for TMJOA treatment.

## 2 Materials and methods

### 2.1 Animals

Female Sprague-Dawley rats (6 and 4 weeks of age) were purchased from the Guangdong Provincial Medical Experimental Animal Center (Guangzhou, China). The experimental procedure was approved by the Animal Care and Use Committee of Southern Medical University. The animals were subjected to light/dark cycles of 12:12 h and a constant temperature of 25°C. *Ad libitum* water and standard chow were provided.

### 2.2 Partial discectomy–induced TMJOA rat model

A discectomy-induced TMJOA rat model was employed in the current study. Twenty-four female Sprague-Dawley rats (6 weeks old) were subjected to surgery and divided randomly into two groups: the surgery group (16 rats) and the sham-operated group (8 rats). The partial TMJ discectomy surgery was performed as previously described (Liu et al, 2021; Hua et al, 2022). Briefly, for the surgery group, following anesthesia with chloral hydrate (10% mL/kg), the joint capsule of the temporomandibular joint was incised, a small T-shaped incision was made in the rat articular disc with a scalpel, and the lateral third of the articular disc was excised to expose the condylar surface. The capsule was incised without exposing or destroying the joint disc in the sham-operated group.

A week post-surgery, the rats were once more randomly divided into two groups (vehicle-treated group and KGN-treated group) and administered intra-articular injections of 50 μL vehicle and KGN (100 nmol·L^-1^) for 8 weeks, respectively. The TMJ condyles of all rats were collected 4 weeks after the last injection for histological and immunohistochemical (IHC) analysis.

### 2.3 Histologic analysis and osteoarthritis research society international (OARSI) histopathological scoring

The TMJ condyles were dissected and isolated. Three independent observers evaluated the macroscopic alterations of the TMJ condyle cartilage using the modified OARSI scoring system (Table 1). The TMJ condyles were then fixed in 4% paraformaldehyde solution for 24 h at room temperature, decalcified for 6 weeks with 10% ethylenediaminetetraacetic acid (EDTA) solution (E1171, Solarbio, Beijing, China) at 37°C, dehydrated in graded ethanol solutions, and embedded in paraffin. The sections were cut using a hard tissue slicer (Leica HistoCore MULTICUT, Germany) along the sagittal plane with a thickness of 4 μm. Hematoxylin and eosin (H&E) and Safranin O/fast green staining were used to stain cartilage tissue sections of the condyles. Three independent observers assessed osteoarthritis severity based on OARSI scoring (Table 2).

**TABLE 1:** Macroscopic semi-quantitative scoring system.

Score	Osteoarthritic damage
0	normal
1	focal surface roughness
2	widespread surface irregularity
3	beginning surface fibrillation
4	severe surface fibrillation
5	beginning erosion
6	severe erosion
7	slight ulceration
8	severe ulceration

**TABLE 2:** OARSI recommended histological scoring system.

Score	Safranin O-fast green staining
0	uniform staining throughout articular cartilage
1	loss of staining in superficial zone of hyaline cartilage <50% the length of the condyle or plateau
2	loss of staining in superficial zone of hyaline cartilage ≧50% the length of the condyle or plateau
3	loss of staining in the upper 2/3’s of hyaline cartilage <50% the length of the condyle or plateau
4	loss of staining in the in the upper 2/3’s hyaline cartilage ≧50% the length of the condyle or plateau
5	loss of staining in all the hyaline cartilage <50% the length of the condyle or plateau
6	loss of staining in all the hyaline cartilage ≧50% the length of the condyle or plateau

### 2.4 Tartrate-resistant acid phosphatase (TRAP) staining analysis

To examine osteoclasts and osteoblasts in the subchondral bone of the TMJ, TRAP staining was performed in accordance with the manufacturer’s protocol (Servicebio, G1050-50T, Wuhan, China). Briefly, paraffin sections of rat TMJ condyles were dewaxed in water, incubated with pure water for 2 h, and then stained with TRAP reagent (37°C, 30 min). Three independent observers counted the number of TRAP-positive osteoclasts at three randomly chosen sites in each sample, and the mean of these three sites was used for statistical analysis.

### 2.5 IHC

Sections of rat TMJ condyles (4 μm thickness) were deparaffinized with xylene, rehydrated with graded alcohol, and then incubated with rabbit primary antibodies against Runx2 (1:500; ab192256; Abcam), Aggrecan (1:400; ab216965; Abcam), Collagen II (1:400; ab34712; Abcam), Sox9 (1:200; ab185966; Abcam), and Ki67(1:300; ab236639; Abcam) overnight at 4°C. After washing with PBS, the sections were incubated with a horseradish peroxidase-conjugated secondary goat anti-rabbit IgG (Goldbridge, Beijing, China) at 37°C for 20 min, and then stained with diaminobenzidine (Goldbridge, Beijing, China). Images were obtained at ×20 magnification with an Aperio Scan Scope slide scanner (Aperio VERSA; Leica Biosystems, United States). The number of positively stained cells at three randomly chosen sites from each sample was counted using the ImageJ software. The average number of positive cells per site was calculated, and the difference between groups was statistically analyzed.

### 2.6 FCSC isolation and culture

FCSCs were isolated from rats according to a previous protocol (Hua et al, 2022). Briefly, the superficial zone tissues of the condyles from 10 rats were carefully separated under a stereomicroscope, and the obtained tissue was cut into small pieces of 1 mm^3^ in a cell culture dish containing PBS, followed by digestion with collagenase I and dispase II (3 mg·mL^-1^ and 4 mg·mL^-1^) at 37°C for 3 h. Single-cell suspensions of FCSCs were cultured (5% CO_2_, 37°C) in basal medium consisting of Dulbecco’s modified Eagle’s medium (DMEM, Gibco) supplemented with 20% fetal bovine serum (FBS, Hyclone), GlutaMAX (Gibco), 1% penicillin/streptomycin (Gibco), and 100 mM 2-mercaptoethanol (Macklin) for 4–6 days. FCSCs were digested with trypsin-EDTA (Gibco) and plated at P1 for *in vitro* experiments.

To assess the effect of KGN on cartilage anabolic factors and catabolic factors, cells were starved overnight in α-MEM containing 1% FBS, then treated for 24 h with 10 ng·mL^-1^ rhTNF-α (peprotech 315-01A) or KGN (100 nmol·L^-1^) alone or in combination.

### 2.7 Multi-lineage differentiation

Multi-lineage differentiation potential of rat FCSCs was tested *in vitro* using chemically defined media for chondrogenesis, osteogenesis, and adipogenesis as previously described (Hua et al, 2022). For chondrogenesis, cells (1× 10^6^ per pellet) were pelleted in 15 mL polypropylene tubes by centrifugation and cultured (5% CO2, 37 °C) for 3 weeks in high glucose Dulbecco’s Modified Eagle medium (Gibco) supplemented with 10^–7^ M dexamethasone, 100 μM L-ascorbic acid, 1% insulin, transferrin, selenium (ITS), 1 mM pyruvate, and 10 ng/mL TGF-β1 (Novoprotein). After 3 weeks, pellets were processed for histology. To induce osteogenesis, FCSCs (5 × 10^4^) were cultured in a 12-well plate for 4–5 weeks in media containing αMEM supplemented with 20% FBS, dexamethasone (10^–8^ M), 100 μM L-ascorbic acid, and 2 mM β-glycerophosphate (Coolaber). To induce adipogenesis, cells (5 × 10^4^) were cultured in a 12-well plate using commercial adipogenic media (BGsciences BGM-1131) for 1 week. Calcium nodules and fat were visualized by staining with alizarin red and Oil Red O, respectively.

### 2.8 Flow cytometry analysis

Cells were incubated for 30 min with or without conjugated specific antibodies at recommended concentrations at 4°C. Then cells were washed, resuspended in PBS, and analyzed on a Flow Cytometer (DxFLEX, Beckman). Unlabeled cells were used as blank control. The antibodies included anti-CD29 (FITC) (102,205, BioLegend), anti-CD45 (Alexa Fluor 647) (202,211, BioLegend), and anti-CD90 (PE) (202,523, BioLegend).

### 2.9 Cell proliferation assay

Following the protocol of cell counting kit-8 (CCK-8) assay (APExBIO, K1081, Houston, United States), the growth of FCSCs in 96-well plates was assessed at 1, 3, 5, 7, and 9 days. FCSCs were seeded into 96-well plates at 2000 cells/well and incubated for 24 h for attachment. KGN (100 nmol·L^-1^) and PBS were added to the media of the experimental and control groups, respectively, and the solution was changed every 2 days. OD values (450 nm) of cell proliferation on days 1, 3, 5, 7, and 9 were measured using a microplate reader.

### 2.10 FCSC pellet culture

A total of 1 × 10^6^ FCSCs were pelleted in a 15-mL polypropylene tube via centrifugation at 2000 rpm for 5 min and then cultured in DMEM supplemented with 10^−7^ mol·L^-1^ dexamethasone, 100 μmol·L^-1^ ascorbic acid, 1% insulin, transferrin, selenium (ITS), 1 mmol·L^-1^ pyruvate, and 10 ng·mL^-1^ TGF-β1 (Novoprotein) for 3 weeks, with medium changes every 2 days.

### 2.11 RNA extraction and quantitative real-time PCR

TRIzol Reagent (AG, AG21102, Hunan, China) was used to extract total RNA from cultured rat FCSCs according to the manufacturer’s instructions. Total RNA (1,000 ng) was reverse-transcribed to cDNA using the Evo M-MLV RT Mix Kit with gDNA Clean for qPCR (AG, AG11728, Hunan, China). Real-time PCR was performed on a PCR instrument (Roche, Lightcycle 96, Switzerland) using SYBR Green Premix Pro Taq HS qPCR Kit II (AG, AG11702, Hunan, China) to analyze SOX9, Aggrecan, Col2a1, and GAPDH mRNA expression, following the manufacturer’s instructions. The relative mRNA expression levels of target genes were normalized to those of GAPDH and then calculated using the 2^−ΔΔCT^ method. The primer sequences used for quantitative real-time PCR are listed in Table 3.

**TABLE 3:** Genes and primer Sequence for real-time quantitative RT-PCR.

Gene	Forward primer sequence (5′to3′)	Reverse primer sequence (5′to3′)
Aggrecan	TTC​CAC​CAG​TGC​GAT​GCA​G	TGG​TGT​CCC​GGA​TTC​CGT​A
Col2a1	CTC​AAG​TCG​CTG​AAC​AAC​CA	GTC​TCC​GCT​CTT​CCA​CTC​TG
SOX9	GAG​CCG​GAT​CTG​AAG​AGG​GA	GCT​TGA​CGT​GTG​GCT​TGT​TC
GAPDH	GCAAGTTCAACGGCACAG	GCC​AGT​AGA​CTC​CAC​GAC​AT

### 2.12 Western blot analysis

FCSCs were lysed in 1x RIPA buffer (Beyotime, P0013B, Shanghai, China) containing 50 mM Tris (pH 7.4), 150 mM NaCl, 1% Triton X-100, 1% sodium deoxycholate, 0.1% SDS, sodium orthovanadate, sodium fluoride, EDTA, leupeptin, 1% protease inhibitor cocktail, and 1% phosphatase inhibitor cocktail (Beyotime, P1005, P1096, Shanghai, China). The BCA Protein Assay Kit (Beyotime, P0012, Shanghai, China) was used to measure the protein concentration in the lysates. Ten micrograms of total protein per sample were loaded in each lane, separated using 10% Precast SDS-PAGE Gel (ACE, Nanjing, China), and transferred to a nitrocellulose membrane (BioRad, United States). After blocking with 1x QuickBlock™ Blocking Buffer for Western blot (Beyotime, P0252, Shanghai, China) for 15 min at room temperature, the membranes were incubated with primary antibodies (SOX9: 1:2000; ab185966; Abcam; Runx2: 1:2000; ab236639; Abcam; GAPDH: 1:5,000; ab37168; Abcam) overnight. The membrane was then washed with TBS-T buffer and incubated with the horseradish peroxidase (HRP)-conjugated secondary antibodies (1:5,000; ab6721; Abcam). After washing, the blots were developed using an enhanced chemiluminescence system (ECL, Merck Millipore, United States). Band density was analyzed using Image Lab Software.

### 2.13 Statistical analysis

Statistical analyses were performed using GraphPad Prism 8 statistical software (GraphPad Inc., La Jolla, CA, United States) or SPSS 22.0 statistical software (IBM, Chicago, IL, United States). Quantitative data are presented as the mean ± SD. One-way analysis of variance (ANOVA) with Tukey’s *post hoc* test was used to determine the statistical significance of differences between three or more groups. At least three repetitions of each experiment were conducted, and *p*-values <0.05 were considered statistically significant. Statistically significant differences are indicated by asterisks (*, *p* < 0.05).

## 3 Results

### 3.1 Intra-articular injection of KGN attenuated cartilage degeneration and subchondral bone resorption in a rat model of partial discectomy–induced TMJOA

**FIGURE 1:**
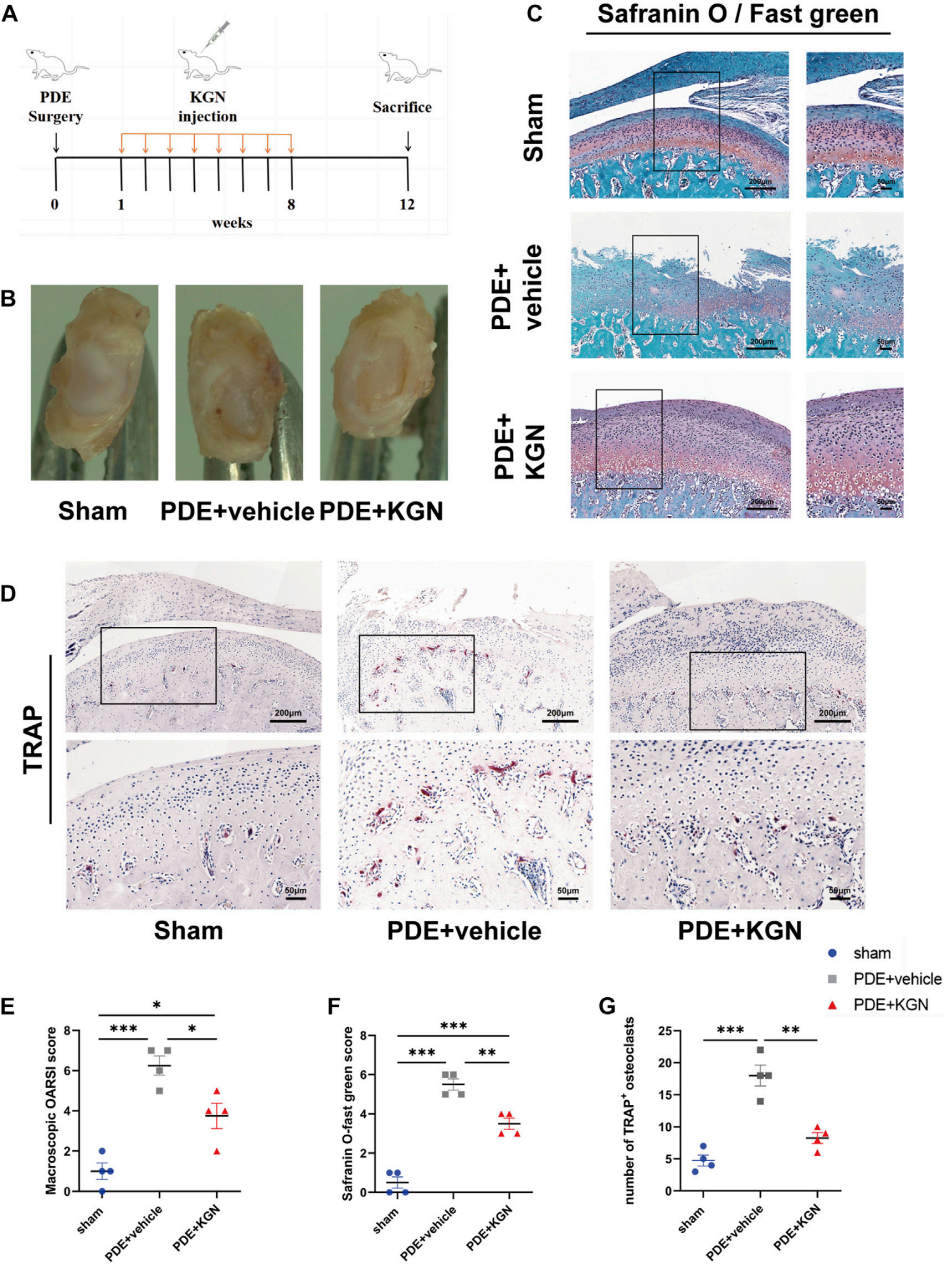
Macroscopic and histopathological evaluation of rat temporomandibular joints (TMJs) after partial discectomy and intra-articular injection of KGN or vehicle. **(A)** A timeline of the *in vivo* study plan. **(B)** Superior view of the TMJ disc and condyle of the sham-operated, vehicle-treated, and KGN-treated groups. **(C)** Representative images of Safranin-O staining of the sham-operated, vehicle-treated, and KGN-treated groups. n = 4 per group. Scale bars = 200 μm (left) or 50 μm (right). **(D)** TRAP staining of TMJ subchondral bone of the sham-operated, vehicle-treated, and KGN-treated groups. n = 4 per group. Scale bars = 200 μm (upper) or 50 μm (lower). **(E)** Macroscopic TMJ condylar cartilage morphology was assessed as per OARSI recommendations. n = 4 per group. **(F)** Quantitative analysis of Safranin O staining in **(C)**. n = 4 per group. **(G)** Quantitative analysis of TRAP-positive osteoclasts in **(D)**. n = 4 per group. Values are presented as the mean ± SD, *p < 0.05; **p < 0.01; ***p < 0.001 between groups indicated by lines.

We first established a partial discectomy-induced TMJOA rat model. A week post-surgery, the vehicle-treated and KGN-treated rats received intra-articular injections of 50 μL vehicle or KGN (100 nmol·L^-1^), respectively, for 8 weeks (Figure 1A). At 12 weeks post-operation, condylar surface injury was more severe in the vehicle-treated group than in the sham-operated group. However, the condylar surface of KGN-treated group rats was smoother than that of vehicle-treated rats, exhibiting no evident damage or irregularities (Figure 1B). The condylar surface morphology of each group was evaluated using a macroscopic semi-quantitative scoring system. The KGN-treated group scored substantially higher than the sham-operated group and significantly lower than the vehicle-treated group (Figure 1E). The sections stained with Safranin O/fast green and TMJ condylar cartilage areas were further assessed using the OARSI histological scoring system. The vehicle- and KGN-treated groups scored considerably higher than the sham-operated group, indicating that the condylar cartilage exhibited evident pathological degeneration at 12 weeks after surgery. However, the KGN-treated group exhibited significantly improved condylar morphology, as indicated by the significantly lower score compared to the vehicle-treated group (Figures 1C,F). TRAP staining was used to examine histopathological alterations in the TMJ condylar subchondral bone of rats. According to our findings, the subchondral bone of the vehicle-treated group contained significantly more TRAP-positive osteoclasts than the sham-operated group, whereas KGN treatment significantly inhibited osteoclast formation 12 weeks post-surgery (Figures 1D,G). These results indicated that intra-articular KGN injection can improve condylar cartilage health and attenuate the abnormal remodeling of the subchondral bone in the partial discectomy-induced TMJOA rat model.

### 3.2 Kartogenin enhanced chondrocyte proliferation, increasing the number of cells in both the superficial and proliferative zones of condylar cartilage *in vivo*

**FIGURE 2:**
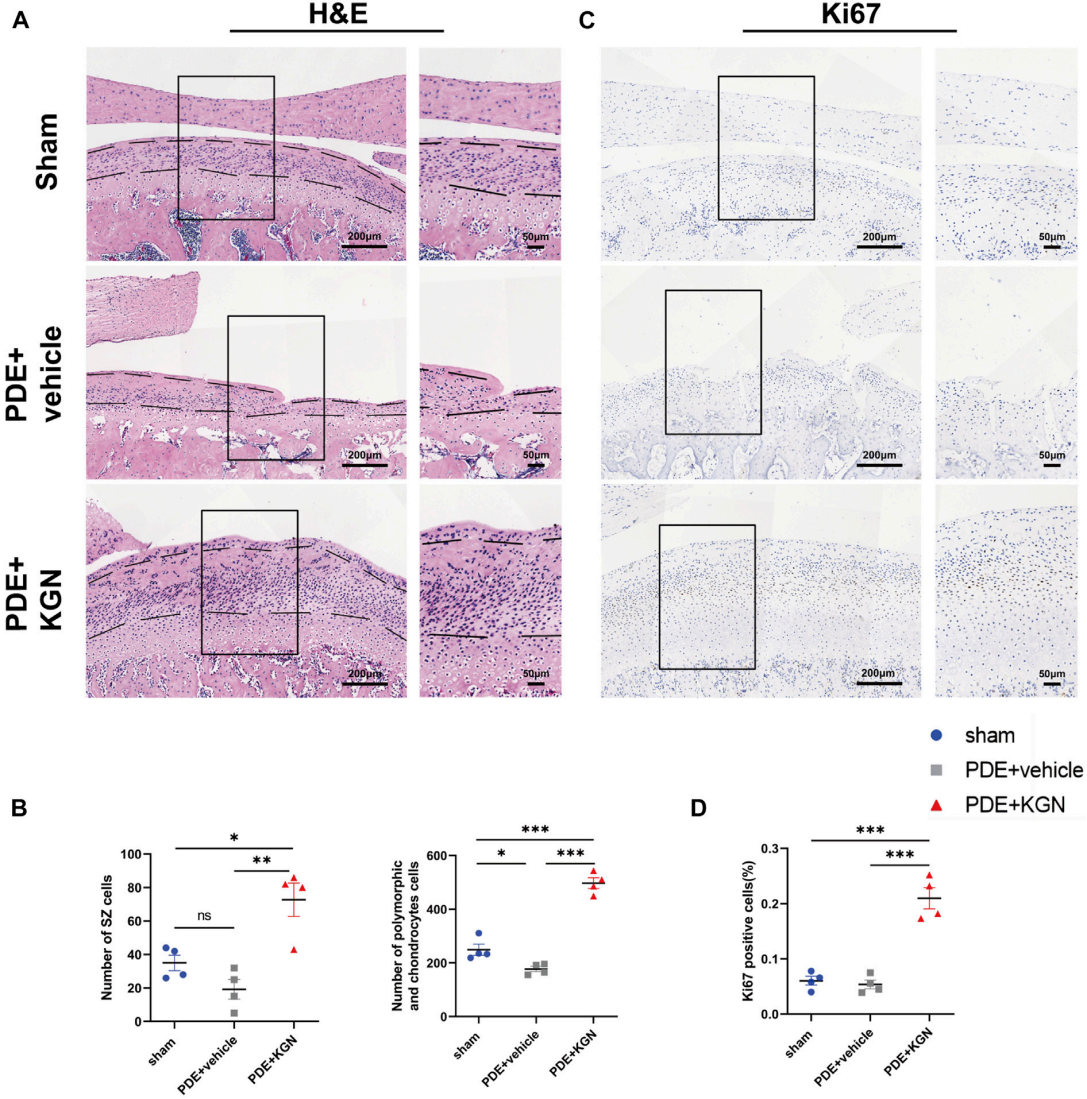
Kartogenin enhanced chondrocyte proliferation and increased the number of cells in both the superficial and proliferative zones of condylar cartilage *in vivo*. **(A)** Representative images of H&E staining of the sham-operated, vehicle-treated, and KGN-treated groups. Scale bars = 200 μm (left) or 50 μm (right). **(B)** The number of nuclei in the superficial zone (SZ) layer, polymorphic layer, and chondrocyte layer of the sham-operated, vehicle-treated, and KGN-treated groups was quantified using ImageJ Software. n = 4 per group. **(C,D)** IHC staining of Ki67 **(C)** of the rat mandibular condyle cartilage and quantitative analysis **(D)** of immunopositive cells of the sham-operated, vehicle-treated, and KGN-treated groups. n = 4 per group. Scale bars = 200 μm (left) and 50 μm (right). Values are presented as the mean ± SD; *p < 0.05, **p < 0.01, and ***p < 0.001 between groups indicated by lines.

Next, we investigated the effect of KGN treatment on the proliferation of condylar chondrocytes by counting the number of nuclei in the superficial zone (SZ), polymorphic, and chondrocyte layers of condylar cartilage via HE staining (Figure 2A). Although the number of nuclei in the SZ layers of vehicle-treated rats was slightly lower than that in the sham-operated group, the number of nuclei in the polymorphic and chondrocyte layers was significantly decreased. Meanwhile, the numbers of nuclei in the SZ, polymorphic, and chondrocyte layers of KGN-treated rats were significantly higher than those in the sham-operated and vehicle-treated groups (Figure 2B). The hypertrophic chondrocyte layer in the KGN-treated group was clearly thicker than that in the vehicle-treated and sham-operated groups when viewed under a microscope. Based on these findings, KGN treatment may promote FCSC proliferation and chondrogenic differentiation in the SZ layer under TMJOA, which in turn increases the number of cells in the polymorphic and chondrocyte layers, thereby promoting the thickening of articular cartilage. IHC was used to analyze the expression of Ki67 in TMJ condylar cartilage so as to determine whether KGN treatment promoted cell proliferation *in vivo* (Figure 2C). Our results showed that, while the expression of proliferation marker Ki67 was slightly reduced in the vehicle-treated group compared to that in the sham-operated group, it was significantly upregulated in the KGN-treated group compared to both the sham-operated and vehicle-treated groups (Figure 2D). These findings implied that KGN therapy promoted condylar chondrocyte proliferation.

### 3.3 Kartogenin promoted FCSC proliferation and the formation of cartilaginous-like tissue *in vitro*

**FIGURE 3:**
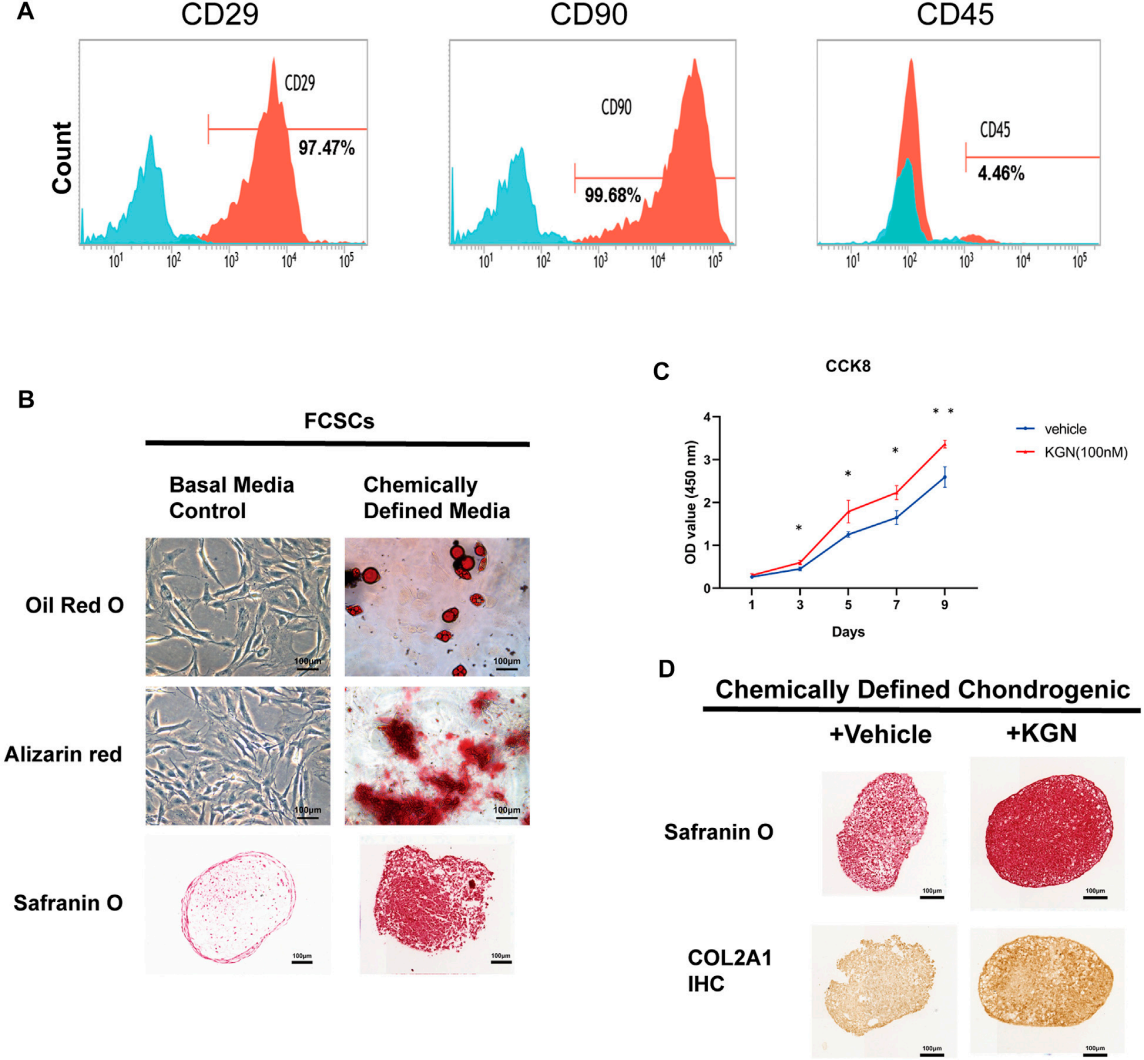
Kartogenin promoted FCSC proliferation and the formation of cartilaginous-like tissue *in vitro*. **(A)** Flow cytometric analysis of cell surface markers in FCSCs. **(B)** Multipotential differentiation of FCSCs *in vitro*. Oil Red O staining of lipid droplets in adipogenic media (top); Alizarin red staining reveals calcium deposition in osteogenic media (middle); Safranin O staining shows chondrogenic potential in pellet cultures (bottom). n = 3 per group. Scale bars = 100 μm. **(C)** The CCK-8 assay was used to determine cell proliferation in each group, and the absorbance at 450 nm was measured. n = 3 per group. Scale bars = 100 μm. **(D)** Safranin O staining (top) and type II collagen (COL2A1) immunostaining (bottom) of FCSCs cultured in chondrogenic medium supplemented with or without KGN. n = 3. Scale bars = 100 μm. Values are presented as the mean ± SD; *p < 0.05, **p < 0.01, and ***p < 0.001 between groups indicated by lines.

We further determined whether KGN treatment could promote the proliferation and differentiation of FCSCs *in vitro*. FCSCs were isolated from the condylar surface of rats, and their capacity for multilineage differentiation was examined *in vitro*. Expression of stem cell surface markers in FCSCs was determined by flow cytometry analysis. The result shown that FCSCs were positive for CD29 and CD90, while negative for CD45 (Figure 3A). This result was consistent with previous finding in rat FCSCs (Embree et al, 2016). Adipogenesis (Oil Red O-positive), mineralization (Alizarin red-positive), and chondrogenesis (Safranin O-positive) were observed in pellet cultures, which indicates that isolated FCSCs can differentiate into multiple lineages (Figure 3B). The CCK8 assay was then used to assess FCSCs proliferation following KGN treatment. Our findings showed that KGN treatment for 1, 3, 5, 7, and 9 days considerably enhanced the proliferation of FCSCs (Figure 3C). A pellet culture system was used to evaluate whether KGN treatment induced FCSC chondrogenesis. Histological analysis using Safranin O staining revealed that the KGN-treated group had greater staining intensity than the control group. Further IHC evaluation also revealed the KGN-treated group had increased type II collagen expression (Figure 3D). These findings demonstrate that KGN treatment promotes FCSCs proliferation and cartilaginous-like tissue formation *in vitro*.

### 3.4 Kartogenin upregulated cartilage anabolic factors while suppressing catabolic factor expression after PDE surgery in rats

**FIGURE 4:**
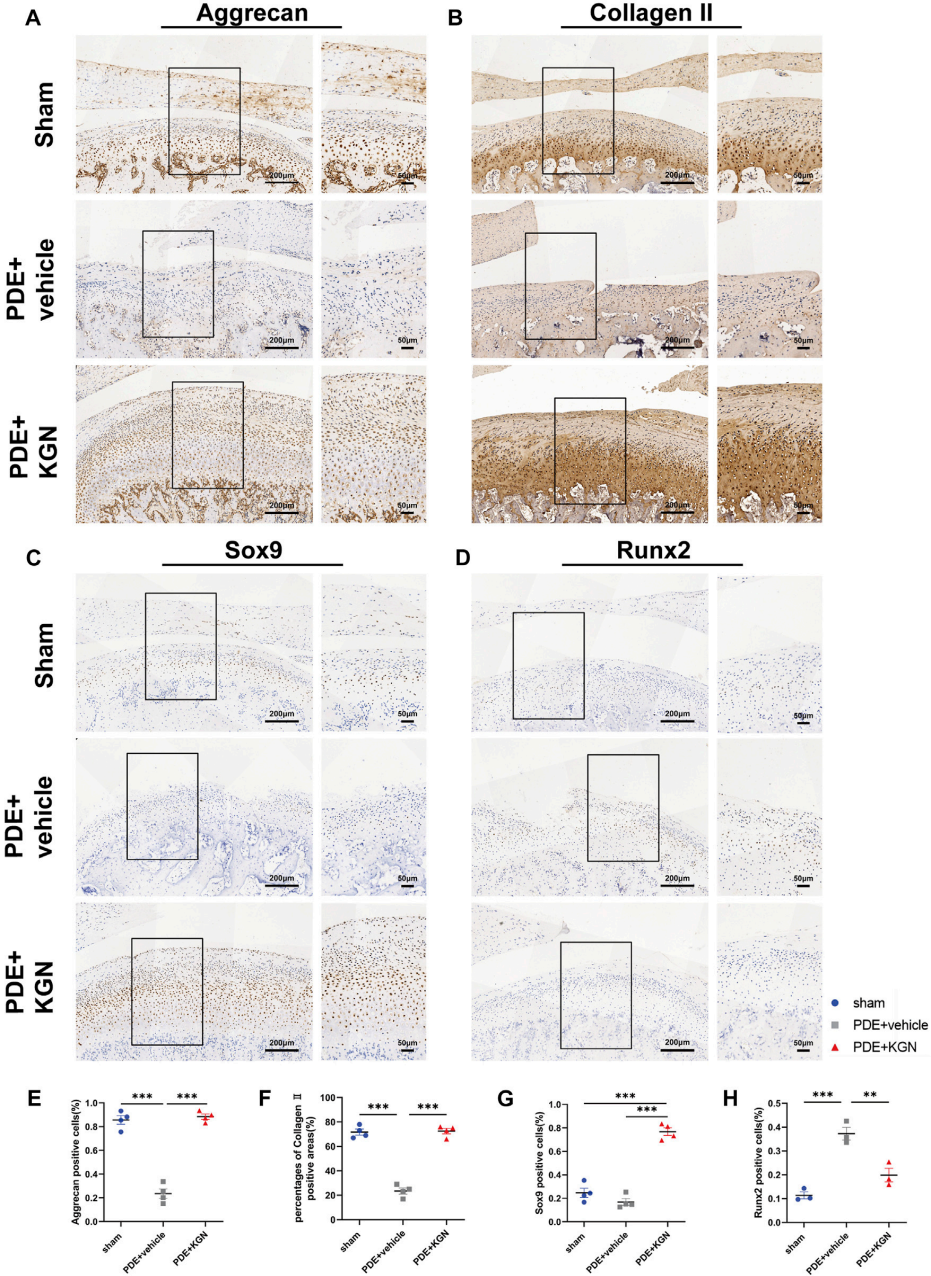
Kartogenin upregulated cartilage anabolic factors while suppressing catabolic factor expression after PDE surgery in rats. IHC staining of Aggrecan **(A)**, Collagen II **(B)**, Sox9 **(C)**, and Runx2 **(D)** of the rat TMJ condyle cartilage and quantitative analysis **(E,F,G, H)** of immuno-positive cells or areas of the sham-operated, vehicle-treated, and KGN-treated groups. n = 4 for Aggrecan, Collagen II, and Sox9; n = 3 for Runx2. Scale bars = 200 μm (left) and 50 μm (right). Values are presented as the mean ± SD; *p < 0.05, **p < 0.01, and ***p < 0.001 between groups indicated by lines.

To elucidate the effect of KGN treatment on the expression of condylar cartilage synthesis- and degradation-related factors in rats, we performed IHC and analyzed the expression of cartilage anabolic factors (Aggrecan, Collagen II, and Sox9) (Figures 4A–C). Our data showed that the percentage of Aggrecan-positive cells and the Collagen II-positive area within the condylar cartilage layer of the vehicle-treated group were significantly lower than in the sham-operated group (Figures 4E–G). However, the expression of these chondrogenesis-associated markers was markedly upregulated in the KGN-treated group relative to the vehicle-treated group. Further, the percentage of Sox9-positive cells was significantly higher than that in the sham-operated group, demonstrating that KGN promotes chondrogenic differentiation and protects condylar cartilage against deterioration. Cartilage catabolic factor Runx2 was significantly downregulated in the KGN-treated compared to the vehicle-treated group while remaining slightly upregulated relative to the sham-operated group (Figures 4D,H). These findings suggest that KGN treatment inhibits chondrocyte hypertrophy in TMJOA.

### 3.5 Kartogenin promoted FCSC chondrogenic differentiation and protected cells from TNF-α–induced inflammatory responses *in vitro*

In osteoarthritis, TNF-α is regarded as the predominant pro-inflammatory cytokine (Alshenibr et al, 2017). We constructed an *in vitro* osteoarthritis model by adding TNF-α to the culture medium (Hua et al, 2022). In order to further investigate the mechanisms by which KGN induces protective effects in TMJ condylar cartilage, we also treated FCSCs with TNF-α in the presence or absence of KGN.

**FIGURE 5:**
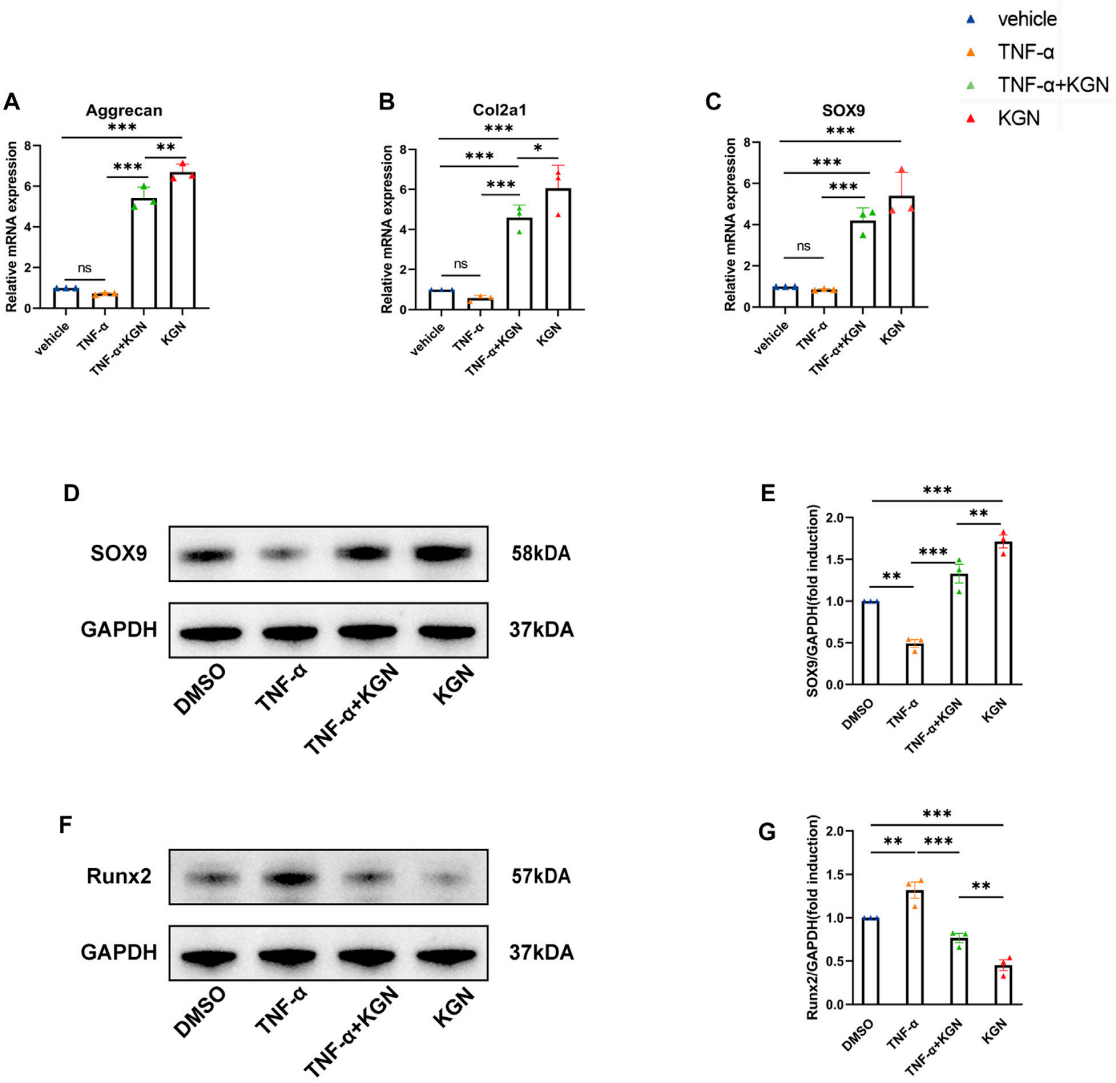
Kartogenin promoted FCSC chondrogenic differentiation and protected FCSCs from TNF-α–induced inflammatory response *in vitro.* FCSCs were treated for 48 h with 10 ng/mL rhTNF-α or 100 nM KGN alone or in combination. **(A,B, C)** Real-time qPCR analysis was performed to evaluate the relative mRNA expression levels of cartilage synthesis-related genes (Aggrecan, Col2a1, SOX9). Gapdh was used as a reference gene. **(D, F)** Western blot analysis of SOX9 **(D)** and RUNX2 **(F)** accumulation in FCSCs of each group. GAPDH was used as the loading control. **(E,G)** Quantification of **(D, F)**. Values are presented as the mean ± SD; *p < 0.05, **p < 0.01, and ***p < 0.001 between groups indicated by lines.

We first examined the effect of KGN treatment on the mRNA expression of genes linked to cartilage synthesis (Aggrecan, Col2a1, and SOX9) in FCSCs (Figures 5A–C). Our findings demonstrated that, in normal conditions, KGN treatment greatly upregulated the mRNA expression of Col2a1, Aggrecan, and SOX9. However, when FCSCs were maintained in inflammatory conditions induced by TNF-α, we discovered that KGN treatment mitigated the TNF-α-induced inhibition of these genes and even upregulated their expression relative to that under normal culture conditions. Subsequently, we performed Western blot analysis for SOX9 and Runx2. Western blot results for SOX9 were in agreement with gene expression data (Figures 5D,E). Runx2 expression was significantly higher in the TNF-α-induced group than in the DMSO group, whereas KGN treatment effectively decreased Runx2 expression under both normal culture and TNF-α-induced inflammatory conditions (Figures 5F,G). These findings suggest that KGN treatment increased the expression of cartilage synthesis-related proteins while protecting FCSCs from TNF-α-induced inflammatory responses.

## 4 Discussion

To our knowledge, this is the first study to evaluate the efficacy of KGN in the treatment of TMJOA. Our results suggest that intra-articular injection of KGN protects condylar cartilage, attenuates aberrant subchondral bone remodeling of the TMJ condyle, and induces condylar cartilage repair and regeneration in the partial discectomy-induced TMJOA rat model. Our data also showed that intra-articular injection of KGN increased the number of FCSCs in the SZ layer as well as the number of chondrocytes in the proliferative/polymorphic and chondrocyte layers, induced thickening of articular cartilage, and protected condylar cartilage against *in vivo* degeneration. In addition, our results indicated that KGN promotes the formation of cartilage-like tissue by TMJ FCSC *in vitro*. Additional cellular and molecular analyses demonstrated that KGN treatment increased the expression of cartilage anabolic factors SOX9, Cox2a1, and Aggrecan in TMJ condylar cartilage *in vivo* as well as in FCSCs *in vitro.*

**FIGURE 6:**
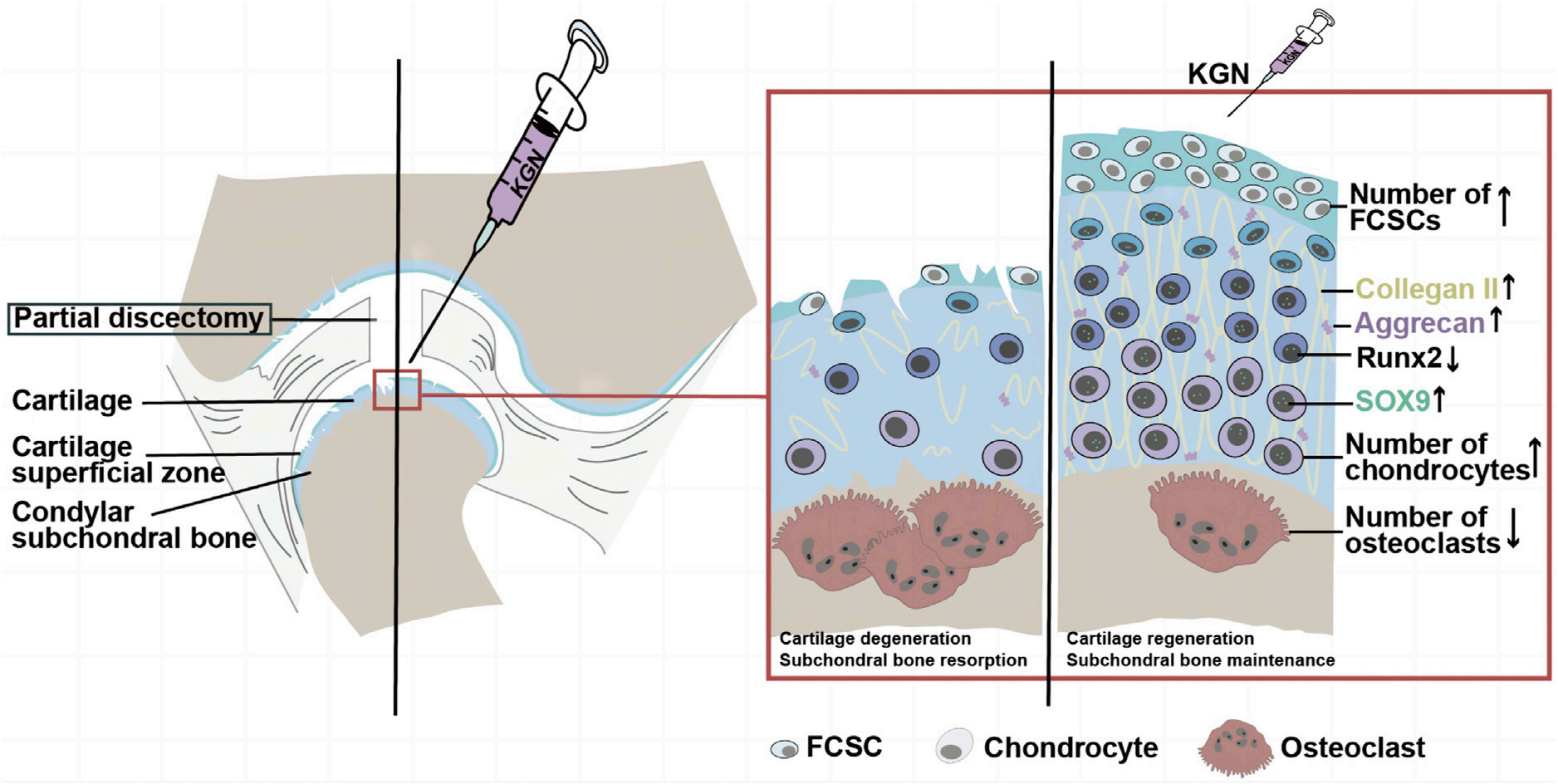
The potential mechanism of KGN-induced protection against TMJ osteoarthritis. During TMJ osteoarthritis, KGN treatment may promote FCSC proliferation and chondrogenic differentiation in the SZ layer under TMJOA, which in turn increases the number of cells in the polymorphic and chondrocyte layers, thereby promoting the thickening of articular cartilage and ultimately encouraging the repair and regeneration of condylar cartilage.

TMJOA is one of the most severe TMJ disorders and is characterized by progressive cartilage degeneration, subchondral bone remodeling, synovitis, and pain. The majority of clinical treatments used today are helpful in reducing pain, and some can even slow joint aging. However, no medication can repair damaged joints (Wang et al, 2015). Previous studies have demonstrated that KGN, a small molecule capable of promoting cartilage differentiation and stem cell proliferation, may be developed as a disease-modifying OA drugs (DMOAD) to treat knee joint osteoarthritis. Moreover, another study revealed that intra-articular injection of KGN into the knee joint can stimulate the proliferation and differentiation of endogenous stem cells on the joint surface, induce articular cartilage regeneration, and promote articular cartilage growth (Liu et al, 2020). Based on our observations in rats, the intra-articular KGN injection induced thickening of the condylar cartilage, protected the condylar cartilage from degeneration, and inhibited abnormal subchondral bone remodeling of the TMJ condylar. We speculate that KGN may also have potential as a DMOAD for the treatment of TMJOA. Other research groups have also demonstrated that endogenous FCSCs localized in the SZ layer of the TMJ condyle have great chondrogenic and osteogenic potential (Embree et al, 2016; Zare et al, 2021). Combined with our findings that intra-articular injection of KGN increases the number of FCSCs in the superficial zone of condylar cartilage, we speculate that intra-articular injection of KGN in the TMJ may promote FCSCs proliferation and chondrogenic differentiation in the SZ layer under TMJOA conditions, which in turn increases the number of cells in the polymorphic and chondrocyte layers, inducing the thickening of condylar cartilage and ultimately encouraging the repair and regeneration of condylar cartilage (Figure 6).

A recent study showed that Runx2 overexpression can accelerate the development of post-traumatic osteoarthritis (Catheline et al, 2019). It has also been suggested that the pro-chondrogenic effects of KGN may be closely associated with the suppression of Runx2 pathways (Jing et al, 2019). Consistent with these findings, our data showed that KGN treatment downregulates Runx2 expression and promotes chondrogenesis under inflammatory conditions both *in vivo* and *in vitro*. Taken together, these results suggest that Runx2 downregulation is a potential mechanism of action through which KGN induces cartilage-protective effects.

It should be mentioned that the purpose of this research was to evaluate the potential of KGN for stimulating the chondrogenesis of endogenous stem cells and the repair of the injured TMJ condyle. The intra-articular injection of KGN into the TMJ used in this experiment may not be the optimal drug delivery system, as, without a scaffold to confine KGN to the injured area, it may induce the overgrowth of surrounding normal tissue (Zhang & Wang, 2014). In addition, due to the rapid metabolism of KGN in the joint space, once-weekly injections are necessary to maintain an effective concentration in the TMJ, an additional limitation of intra-articular injections (Xu et al, 2015; Hu et al, 2017). Fortunately, intra-articular drug delivery systems, such as liposomes, hydrogels, nanoparticles, and microparticles, have been utilized for prolonged and sustained drug release in joints. Owing to their superior biological properties, these delivery systems enable KGN to continuously and safely exert its effects within the damaged TMJ condyle, thus leading to a greater therapeutic benefit relative to intra-articular injection (Xu et al, 2021; Yu et al, 2021; Zhang et al, 2022). KGN can therefore be adapted into a slow-releasing, small-molecule DMOAD to be injected for TMJOA treatment in humans.

## 5 Conclusion

Taken together, our results demonstrate that KGN treatment induces the thickening of condylar cartilage and ultimately promotes the repair and regeneration of damaged condylar cartilage. Therefore, KGN has potential as a therapeutic agent for TMJOA. Extensive studies are still required in order to confirm the specific regulatory mechanism of KGN as well as its biosafety for TMJOA treatment.

## Data Availability

The original contributions presented in the study are included in the article/supplementary materials, further inquiries can be directed to the corresponding author.

## Abbreviations

KGN, kartogenin; TMJ, temporomandibular joint; TMJOA, temporomandibular joint osteoarthritis; FCSC, fibrocartilage stem cell; MSC, mesenchymal stem cell; OARSI, osteoarthritis research society international; EDTA, ethylenediaminetetraacetic acid; H&E, hematoxylin and eosin; TRAP, tartrate-resistant acid phosphatase; DMEM, Dulbecco’s modified Eagle’s medium; CCK-8, cell counting kit-8; HRP, horseradish peroxidase.
